# Coordination of cardiac rhythmic output and circadian metabolic regulation in the heart

**DOI:** 10.1007/s00018-017-2606-x

**Published:** 2017-08-21

**Authors:** Paishiun Nelson Hsieh, Lilei Zhang, Mukesh Kumar Jain

**Affiliations:** 1 Department of Medicine, Case Cardiovascular Research Institute, Case Western Reserve University, 2103 Cornell Road, Room 4-503, Cleveland, OH USA; 2 Harrington Heart and Vascular Institute, University Hospitals Case Medical Center, Cleveland, OH USA; 3 Department of Pathology, Case Western Reserve University, Cleveland, OH USA; 4 Department of Molecular and Human Genetics, Baylor College of Medicine, Houston, TX USA

**Keywords:** Cardiac, Cardiovascular, Metabolism, Peripheral clock, Circadian, Transcriptomics

## Abstract

Over the course of a 24-h day, demand on the heart rises and falls with the sleep/wake cycles of the organism. Cardiac metabolism oscillates appropriately, with the relative contributions of major energy sources changing in a circadian fashion. The cardiac peripheral clock is hypothesized to drive many of these changes, yet the precise mechanisms linking the cardiac clock to metabolism remain a source of intense investigation. Here we summarize the current understanding of circadian alterations in cardiac metabolism and physiology, with an emphasis on novel findings from unbiased transcriptomic studies. Additionally, we describe progress in elucidating the links between the cardiac peripheral clock outputs and cardiac metabolism, as well as their implications for cardiac physiology.

## Introduction

Over millennia, repeated cycles of day and night have led to the evolution of biologic clocks which keep time with the rising and setting of the sun. In mammals, the suprachiasmatic nucleus (SCN) functions as a central pacemaker, receiving input from environmental cues to align physiological rhythms throughout the rest of the body. Within the SCN, a transcriptional/translational feedback loop (the molecular clock) entrained by light sustains oscillations in gene expression in a diurnal fashion. This clock is coupled to many other cellular processes, conferring rhythmicity to critical functions such as metabolism. However, major organs, such as the liver and heart, have been shown to harbor independent rhythms; these tissue-intrinsic clocks are termed peripheral clocks and generally cycle in rhythm with the central clock [1, 2]. Peripheral clocks, in contrast to the central clock, are sensitive to other environmental cues (zeitgebers), such as diet and temperature. In fact, the peripheral clock in the liver can be desynchronized from the SCN by reverse-feeding regimens, shifting phase by approximately 9–12 h [3, 4]. Diet has a similar effect in other metabolic organs such as skeletal muscle and the heart.

The mechanism by which different organs achieve rhythmicity in diverse tissue and cellular functions is a central question in circadian biology. The central clock in the SCN has powerful influences on circadian rhythms of the organism, and it has been hypothesized that rhythmicity is achieved through circadian fluctuations in autonomous nervous system and circulating hormones [5]. In the heart, diurnal oscillatory stimuli have profound influences on cardiomyocyte function. For example, in humans, the renin–angiotensin–aldosterone system has been found to oscillate, while evidence in rats has demonstrated diurnal variation in vasoactive intestinal peptide and atrial natriuretic peptide [6–9]. Interestingly, clock genes continue to oscillate in ex vivo hearts, suggesting that time-of-day dependence of cardiac-specific functions is also mediated through intrinsic mechanisms, i.e., the peripheral clock [10]. This is further supported by the loss of rhythmicity of cardiac physiology and gene expression in the cardiac-specific mutant mice of a core clock component, such as CLOCK and BMAL1 (dominant negative cardiomyocytes-specific *Clock* mutant, CCM and cardiomyocytes-specific *Bmal1* knockout, CBK mice, respectively), as discussed in more detail later [11].

For decades, the heart has been noted to demonstrate diurnal variation in critical physiological and pathological functions [12–14]. More recently, diurnal variation in fuel utilization and metabolism has gained attention in circadian biology in several organs due to the consequences of its disturbances [15–17]. This review will focus on the diurnal variation of cardiomyocyte metabolism controlled by the cardiac peripheral clock and how time-of-day alterations in metabolism manifest in cardiac circadian physiology. We will emphasize the recent progress using unbiased genome-wide surveys in elucidating the molecular links coupling circadian mechanisms with metabolism and cardiac physiology. However, our investigation of the interplay between the peripheral clock, metabolism, and ultimately physiology remains in its infancy [18].

## The mammalian molecular clock

**Fig. 1:**
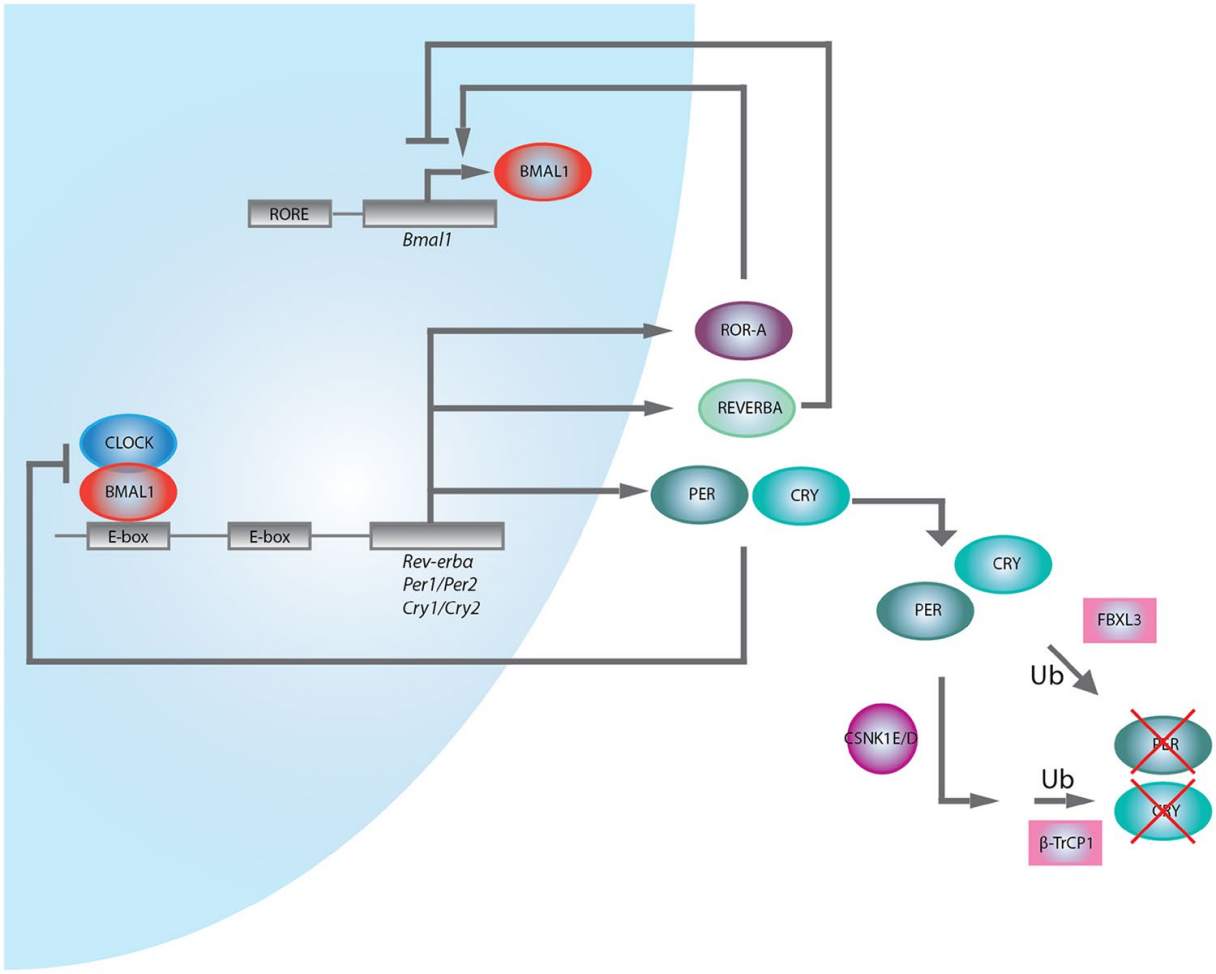
The molecular clock. A transcriptional/translational feedback loop maintains cellular rhythmicity and is entrained to external cues such as light and diet. *Ub* ubiquitinylation

Whether located centrally or peripherally, cell-autonomous rhythms in mammals are primarily maintained via a self-sustaining transcriptional/translational feedback loop which is present in every cell [19]. The components of this core “molecular clock” have been well studied in various cell types and demonstrate little variation [20]. CLOCK and BMAL1 heterodimerize and bind promoter E-box elements to activate transcription of *Cryptochrome* (*Cry*) and *Period* (*Per*) genes. Sufficiently high levels of PER and CRY lead to their nuclear translocation and interaction with the CLOCK:BMAL1 heterodimer to inhibit further transcription. The CLOCK:BMAL1 heterodimer also regulates the nuclear hormone receptors REV-ERBα and β and RAR-related orphan receptor α (ROR-α), which repress and activate CLOCK:BMAL1 expression, respectively [21]. REV-ERBα and ROR-α have both been shown to regulate *Bmal1* via binding to retinoic acid-related orphan receptor response elements (ROREs) present in the *Bmal1* promoter (Fig. 1) [22].

The control of the core clock is also subject to epigenetic regulation, for example, histone acetylation, histone methylation, histone ubiquitination, DNA methylation, chromatin looping, etc. This topic has been recently reviewed in detail by Papazyan et al. [23]. In particular, circadian transcription coupled to histone acetylation as mediated by histone acetyltransferases (HATs) and histone deacetylases (HDACs) is the most studied [24–26]. Rhythmic changes in *Per1* and *Per2* expression in mouse fibroblasts and liver cells positively correlate with acetylation of histones H3 and H4, while treatment with the HDAC inhibitor trichostatin A induces *Per1* expression and acetylation of histones H3 and H4 [27]. Additionally, the histone acetyltransferases p300/CBP, PCAF, and ACTR have been shown to associate with CLOCK and NPAS2 to regulate clock gene expression, and REV-ERBα-mediated repression of target genes is partially dependent on recruitment of HDAC3 via the nuclear receptor corepressor (NCoR) [28, 29]. In fact, CLOCK itself exhibits histone acetyltransferase activity, a direct link between the core clock machinery and chromatin remodeling [30]. Across the genome, K3K9 and H3K27 acetylation occupancy, as well as occupancy of several other chromatin modifications, is circadian at transcription start sites in genes being expressed, providing evidence of broad chromatin remodeling by the mammalian clock [31]. Other chromatin marks such as histone methylation and ubiquitination, as well as three-dimensional chromatin architecture contribute to circadian gene expression, and these have been extensively reviewed [23].

Finally, the core clock components are also influenced by post-translational modifications. PER and CRY proteins can be phosphorylated by casein kinase Iε and δ, which may target them for ubiquitination and subsequent degradation, or directly ubiquitinated via the β-TrCP1 and FBXL3 E3 ubiquitin ligases [32–34].

## Known circadian CV physiology

The diurnal rhythmicity in the physiological parameters (heart rate, blood pressure, QT interval, etc.) and pathological conditions of the cardiovascular system (arrhythmia, sudden cardiac death, myocardial infarction, aneurysm rupture, exacerbation of heart failure, stroke, etc.) have been recognized for decades. Cardiac functioning and physiology are under the complex control of a wide range of intrinsic and extrinsic factors. For example, blood pressure variation may result from cyclic physical activity as shown by studies in shift workers. Endogenous factors contribute via neural hormonal systems. However, the best evidence of an intrinsic circadian control of BP comes from animal studies with CCM or CBK mice and carefully designed human studies [35, 36].

The function and the physiology of the heart are intimately linked to cardiac metabolism. For example, substrate availability varies in a 24-h day, which directly affects cardiac function. Conversely, cardiac demand fluctuates in a day–night cycle and failure to match it with appropriate metabolic activity will lead to cardiac dysfunction as well. In this section, we will discuss several well-studied examples of cardiac physiology with an emphasis on their interactions with metabolism.

### Contraction

**Fig. 2:**
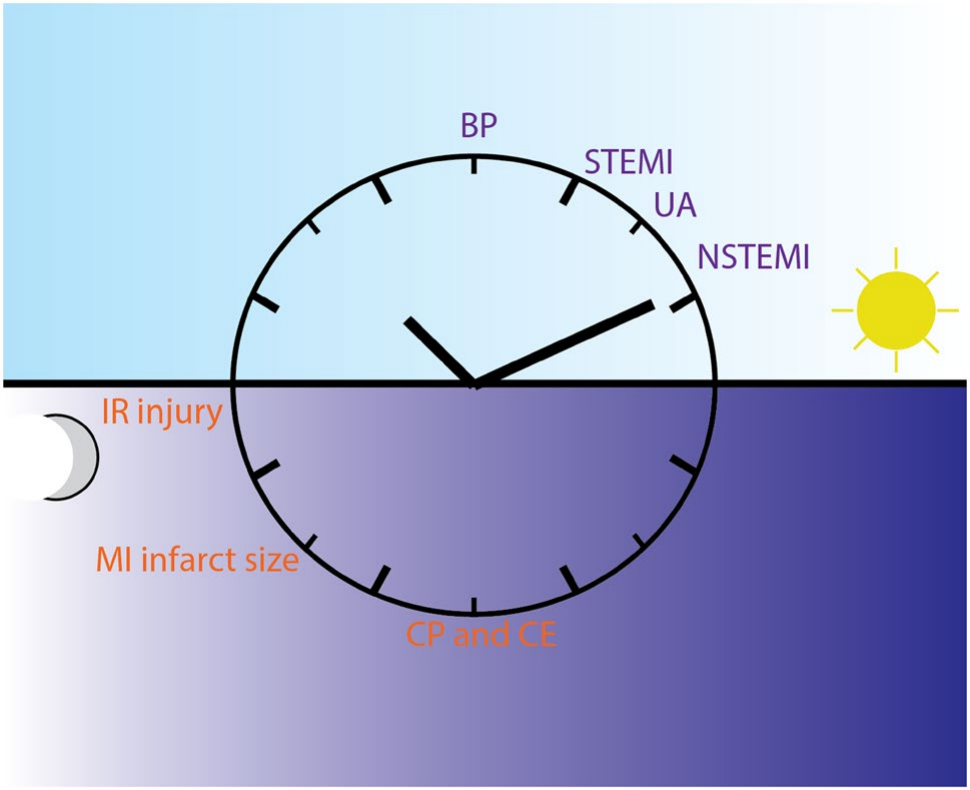
Cardiac circadian physiology and pathology. Many cardiac physiologic functions as well as cardiovascular pathologies exhibit time-of-day dependency in humans, especially in the early morning hours. *BP* blood pressure, *STEMI* ST elevation myocardial infarction, *UA* unstable angina, *NSTEMI* non-ST-elevation myocardial infarction, *IR injury* ischemia–reperfusion injury, *MI infarct size* myocardial infarct size, *CP and CE* cardiac power and cardiac efficiency. *Dark blue font* human events, *orange font* mouse events. Of note, mice are nocturnal and, therefore, relative to their resting/active cycle, many physiologic events occur for mice at approximately opposite chronologic time as for humans

Rat cardiomyocytes have greater intracellular calcium levels and basal contraction when isolated during resting phase than active phase. The β-agonist, isoproterenol, induced calcium spike is also greater during the resting phase, reflecting a larger calcium pool in the sarcoplasmic reticulum [37]. In wild-type mouse hearts, there is a similarly marked diurnal variation in response to increasing work load ex vivo, with higher increase in cardiac power and efficiency during the active phase. This diurnal variation is abolished in CCM mice hearts (Fig. 2) [38]. Further, mice in a model of cardiac hypertrophy via transverse aortic constriction exhibited altered cardiac left ventricular dimensions and decreased fractional shortening when subjected to 20-h (10 h light, 10 h dark) light cycles to disrupt rhythms compared to mice on a 24-h cycle [39]. Although myosin expression is not circadian, myosin sensitivity to Ca^2+^ is rhythmic in wild-type mice, a rhythmicity which is abolished in CCM mice. Further, phosphorylation of myofilament proteins such as tropomyosin and cardiac myosin binding protein-C was found to exhibit diurnal variation which was lost in CCM mice [40].

In vivo data, however, are not available, as they will be subjected to a change in load, which also varies in a diurnal fashion. Blood pressure (BP) shows a day–night cycle, with a peak in the mid-morning and a trough at night. Normal ambulating humans have a 10% variation between the peak and the trough [12]. The diurnal variation in BP results from both cyclic physical activity and other endogenous factors such as autonomic tone and humoral factors, including the renin–angiotensin–aldosterone axis and cortisol [41, 42]. Heart rate varies in mammals as well; mathematical modeling of heart rate fluctuations across the diurnal cycle has demonstrated scale-invariant patterns in rats and humans, implying an intrinsic control mechanism common in mammals [43]. Additionally, both CCM mice and CBK mice demonstrated a slowed heart rate and loss of circadian rhythmicity; in particular, in CCM mice, a larger decrease in heart rate during the dark phase contributed to the loss of circadian rhythmicity [38, 44]. The diurnal variability in contractility necessitates a plastic metabolic program that can match its demand (Fig. 2).

### Myocardial infarction

The accurate timing of the onset of myocardial infarction (MI) is challenging in human studies. Although the onset of MI is demonstrably circadian, the pathobiology underlying circadian occurrence of vulnerable plaque rupture remains complex and likely involves circadian rhythmicity of several factors including BP, platelet aggregation, and fibrinolysis. A large study of 5645 individuals from 8 populations over a median follow-up of 11.4 years found that high morning systolic blood pressure surge could be a useful prognostic factor in stratifying cardiovascular risk [45]. This is consistent with the observation that a high rate of variability in systolic BP is correlated with increased medial hypertrophy of the large arteries [46]. Additionally, the morning blood pressure surge may directly contribute to destabilizing plaques; compared with plaques from individuals with hypertension but lacking a morning BP surge, plaques from individuals with a morning BP surge displayed an unstable plaque phenotype, with increased infiltration by immune cells (macrophages, T lymphocytes), increased ubiquitin–proteasome activity, nuclear factor-κB, matrix metalloproteinase-9, and less collagen content [47].

The influence of circadian variation in inflammatory response on circadian MI deserves special mention. Ly6C^hi^ inflammatory monocytes demonstrate diurnal variation in trafficking to sites of inflammation, a phenotype abolished by deletion of *Bmal1*, and myeloid-specific deletion of *Bmal1* exacerbates diet-induced obesity and insulin resistance in mice, presumably due to alterations in chronic inflammatory responses [48]. External circadian signals from the autonomic nervous system also influence variable expression of ICAM-1 and CCL2 in the endothelium of skeletal muscle and VCAM-1 and CXCL12 in the bone marrow to influence leukocyte emigration to tissues [49]. Additionally, tumor necrosis factor α and interleukin-6 secretion after lipopolysaccharide stimulation is circadian, observations in line with the finding that macrophages have an endogenous clock with about 8% of transcripts showing rhythmic expression (including components involved in LPS-induced responses such as nuclear factor κB, JUN, and FOS), suggesting that beyond circadian recruitment of inflammatory cells, local inflammatory responses once they have arrived remain rhythmic [50]. Indeed, Schloss et al. show in a recent study that cardiac neutrophil recruitment, myeloid progenitor production, expression of cardiac adhesion molecule, and chemokine levels are all increased specifically during the active phase, and that mice subjected to left anterior descending coronary artery ligation during this phase develop larger infarcts and worsened cardiac function compared to control mice operated upon during the rest phase. Blockade of CXCR2 by antibody selectively reduced cardiac infiltration by neutrophils in the active phase but not the rest phase [51].

Platelet aggregability, circulating platelet aggregates, and fibrinolytic activity also vary in a circadian fashion, associating with a well-known early morning hypercoagulability and elevations in tissue factor pathway inhibitor and activated factor VII levels [52–55]. Platelet aggregability in the morning has been associated with risk of MI and sudden cardiac death [56]. In contrast, fibrinolytic activity is highest in the afternoon and lowest in the morning, in accordance with observations that tissue plasminogen activator inhibitor 1 levels are highest in the early morning, leading to lower tissue-type plasminogen activator [52, 57, 58]. Low morning fibrinolytic activity has been proposed as an independent risk factor for first acute MI in men and women [59]. Together, these studies provide evidence that there is a complex interplay of circadian factors underlying the time-of-day dependence of MI.

The first study to demonstrate an early morning clustering of MI used the time of the first elevation in the plasma creatine kinase MB level to estimate the time of the onset [60]. Subsequent studies, including data from the National Cardiovascular Data Registry, TIMI III registry and TIMI IIIB trial chose to use the onset of symptoms and confirmed the excess of STEMI, unstable angina, and non-Q MI in the early morning hours [61, 62]. Further, it has been shown that this chronological effect can be ameliorated by either a beta blocker or aspirin, suggesting diurnal variation in adrenergic activity and platelet aggregation play important roles (Fig. 2) [60, 63].

Although it is generally accepted that the timing of the MI has a circadian rhythmicity, there is some controversy regarding the size of the MI. Durgan et al. demonstrated that wild-type mouse hearts subjected to ischemic/reperfusion (IR) injury at the sleep to wake transition (ZT12) have a 3.5-fold increase in infarct size compared to the wake to sleep transition, and this variation was abolished in CCM mice [64]. This provided the first evidence that there is a time-of-day-dependent susceptibility to IR injury that is cell autonomous to the cardiomyocytes. However, Eckle et al. found a reduction in infarct size at ZT12 and ZT18 compared to ZT0. The same group also reported that mPer2 (dominant negative) mutant mice have larger infarct sizes and loss of ischemic cardioprotection when compared to the wild type. They demonstrated that PER2 is a downstream target of adenosine receptor A2b, which leads to stabilization of PER2 during myocardial ischemia and subsequent stabilization of hypoxia-inducible factor-1α and induction of glycolysis. Stabilization of PER2 by intense light can bypass A2b signaling and induce glycolysis and cardioprotection during ischemia [65]. The exact same mutant mice mPer2 were also studied by Virag et al., who showed that mPer2 mice have reduced infarct sizes after non-reperfused MI, after IR, and after preconditioned IR. More studies by different groups are required to elucidate the reasons for the discrepancy [66, 67]. Recently, Rotter et al. demonstrated IR tolerance was greatest in hearts at the transition from wake to rest, and that this tolerance was dependent on protein regulator of calcineurin 1 (*Rcan1*), whose protein levels oscillate in a circadian fashion and peak at this transition [68]. Nonetheless, the variable susceptibility to ischemic injury suggests a dynamic cellular metabolic and reduction/oxidation environment (Fig. 2). Several initial studies in humans investigating the relationship between time of symptom onset and infarct size in STEMI achieved varying results and were the subject of debate [69–72]. However, more recent studies, including a large study of 6223 STEMI patients treated with primary angioplasty in Switzerland using peak creatine kinase as a measure of infarct size, support the conclusion that myocardial infarct size as well as in-hospital mortality exhibit circadian variation [73–75].

### Drug targets

Zhang et al. recently looked at the top 100 best-selling drugs in the US and found that 56 targets (directly bound and affected by the drug) are encoded by a circadian gene, which oscillates at the transcript level [76]. 23 of these 56 drugs have a plasma half-life of less than 6 h, suggesting the time of administration is critical to allow effective interaction with the targets. However, the importance of the chronality of very few of these medications is appreciated. In the list, several widely used cardiovascular medications are included, such as Diovan and Toprol XL. Brand names such as “XL” are used to suggest “extended release” and “long acting”, which may be misleading. Interestingly, the primary target for aspirin, *Ptgs1* (COX1, cyclooxygenase-1) oscillates in the heart, lung, and the kidney. Aspirin as a standard therapy for myocardial infarction has a biological half-life of 2–3 h; therefore, given the circadian rhythm of the onset of heart attacks, the optimal window for cardioprotection is fairly narrow. This window of benefits also coincides with previously reported clinical studies that nighttime aspirin is more effective in reducing ambulatory blood pressures [77, 78]. Studies by Hermida et al. showed that the effectiveness of antihypertensives also varies depending on time of day. They found a strong association between reduced BP during sleep, or “dipping”, and ramipril 5 mg daily taken at bedtime as opposed to taking the drug upon awakening, and an increased proportion (from 43 to 65%, *P* = 0.019) of patients with controlled ambulatory BP at 6 weeks [79]. In the Heart Outcomes Prevention Evaluation (HOPE) study, ramipril administered at night provided benefit three times greater than predicted based solely on BP reduction [80]. A separate study in 2156 individuals with a mean follow-up of 5.6 years, the Ambulatory Blood Pressure Monitoring for Prediction of Cardiovascular Events (MAPEC), concluded from comparing morning and nighttime doses that individuals on nighttime dosing had overall better BP control and that this was associated with lower risk of total cardiovascular disease events (relative risk 0.39, confidence interval 0.29–0.51, *P* = 0.001) [81, 82].

Although most of the pharmacokinetics occur outside the cardiovascular system itself, our understanding of the chrono-metabolism of these drugs and the chronality of their targets will further improve the efficacy and reduce side effects of these already widely used medications.

## Cardiac rhythmic output

Rhythmic cardiac physiology serves as the integrated readout of both rhythmic neurohormonal regulation derived from the central clock and rhythmic gene expression originating from the peripheral clock. The neurohormonal regulation has long been characterized and, thus, in this section, we will focus on the recent progress in characterizing the role of the peripheral clock in the heart.

### Transcriptomics

The core clock machinery is essentially the same in every cell, yet cells are able to limit rhythmicity at the gene expression level to a particular subset (3–10%) of the transcriptome relevant to the distinct function of the organ in which they reside. This was first demonstrated by the pioneering work of Panda et al. [83]. A recent study by Zhang et al. further substantiated this observation by high-chronological-resolution transcriptomic studies across 12 mouse organs, where they demonstrated that the steady-state RNA level of 43% of mouse protein-coding genes cycle in at least one of the 12 organs examined [76]. This allowed them to suggest that more than half of the protein-coding genome is rhythmic somewhere in the mammalian body at the transcript level.

Panda et al. have predicted that such tissue-specific temporal organization demands the existence of “slave oscillators”, or factors whose rhythmicity is driven by the cell-autonomous core clock and whose expression or activity is tissue specific, allowing the clock in disparate peripheral areas to selectively confer or suppress rhythmicity to the necessary cellular functions unique to that tissue’s function [83]. Although this concept has been proposed for more than a decade, understanding in “slave oscillators” is still poor. Further, although studies have been conducted to search for modifiers of the circadian regulation, most attention has been focused on the “positive” regulators, which promote oscillation, change the duration of the period or increase amplitude. Little efforts have been bestowed upon the “negative” regulators, which inhibit oscillation or reduce amplitude. Conceptually, negative regulators are equally important in attaining this tissue-specific landscape of temporal transcriptomic control.

Similarly, transcriptomic studies in the heart have provided crucial insights into understanding circadian regulation. Since the earliest study by Storch et al., multiple groups have used oligo microarray and RNAseq to characterize the oscillating genes in the heart [76, 84, 85]. It is generally agreed that like other well-characterized tissues, ~10% of the genes expressed in the heart oscillate. Young et al. reported microarray analysis of CBK mice, which allowed identification of BMAL1-dependent oscillating genes and subsequent comparisons to pre-existing system-level transcriptional regulatory databases including gene expression, presence of a putative BMAL1/CLOCK binding site, and prior confirmed BMAL1 binding in the regulatory region in mouse livers via ChIPseq (chromatin immunoprecipitation and deep sequencing), to identify direct BMAL1 targets in the heart and found 19 putative BMAL1 target genes of which 15 of these were also differentially expressed in CCM mice, suggesting they are highly likely to be bona fide targets for BMAL1. These targets include several relevant metabolic genes such as *Nampt* and *Dgat2*, highlighting the direct regulation of core clock machinery on metabolic targets [38, 86].

More recently, we demonstrated the mechanistic principle of “positive” and “negative” regulation in circadian gene regulation by studying the cardiac-specific null mice of transcription factor KLF15 (Kruppel-like factor 15), which is a direct target of BMAL1 in the core clock machinery [85, 87]. Using transcriptomic approaches in the heart in a circadian fashion, we showed that Kruppel-like factor 15 (KLF15) directly regulates large numbers of oscillatory genes in the heart (75% of all genes). Importantly, this study provided the first evidence for negative regulation by KLF15 through engaging the circadian repressor REV-ERBα [85]. This prevents aberrant fluctuation of genes, which may oscillate in other tissues or organs. KLF15 is likely a bona fide “slave oscillator” in the heart, for which it not only promotes oscillation but also suppresses oscillation. This corroborates and extends the original concept of the “slave oscillator”.

**Fig. 3:**
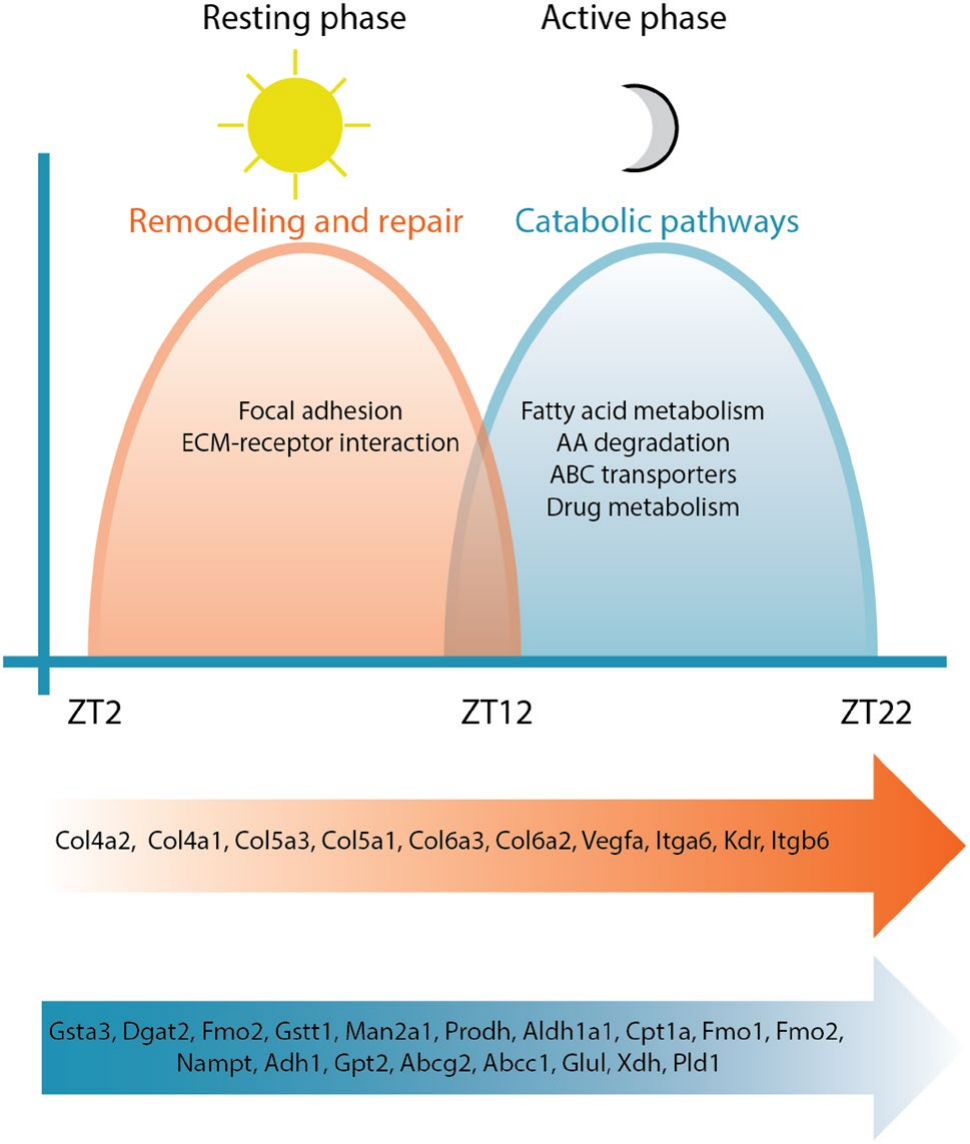
Circadian variation in transcriptional outputs. In experimental mouse models, the peripheral clock in heart coordinates responses in diverse transcriptional programs to anticipate diurnal variation in cardiac demands and maximize efficiency

Another key observation is that we demonstrated a temporal organization of oscillating transcripts, with a night peak (active phase) coinciding with activation of catabolic pathways and a day peak (resting phase) coinciding with remodeling and repair processes [85]. This underscored that optimizing energy metabolism is a key aspect of cardiac circadian regulation. In contrast to studies in the mouse liver, where biosynthetic pathways often dominate, oscillating genes in the heart with peak expression during the active phase are enriched for catabolic pathways, including fatty acid degradation, amino acid metabolism (branched chain amino acid, tryptophan, lysine, and tyrosine), carbon metabolism (genes in the TCA cycles), ABC transporters, and drug metabolism (cytochrome P450). This is consistent with the heart as the largest energy consumer in the body and the active phase is when eating as well as physical activity occur (Fig. 3).

Among the metabolic enzymes that were found to be oscillating, several of them share an interesting connection with nicotinamide adenine dinucleotide (NAD+), which include the rate-limiting enzyme in the NAD(+) biosynthesis salvage pathway, nicotinamide phosphoribosyltransferase (NAMPT), the mitochondria oxidation–phosphorylation enzyme, succinate dehydrogenase (SDHa), and the mitochondria tricarboxylic acid cycle enzyme, isocitrate dehydrogenase (IDH3g). Regulated by the core clock machinery, cellular levels of NAD(+) oscillate in a circadian fashion; indeed, CLOCK binds rhythmically to the *Nampt* promoter to influence its expression [88, 89]. NAD(+) is a critical co-enzyme of many cellular enzymes and its levels in turn influence many cellular processes. Sirtuins are NAD(+)-dependent protein deacetylases, which are involved in a broad array of cellular activities, including cellular metabolism. In the liver, Sirtuin 1 (SIRT1) is required for the high-magnitude oscillation of several core clock genes by promoting the deacetylation and degradation of PER2 and BMAL1, thus coupling energy metabolism with the circadian clock [90, 91]. SIRT1 can further influence metabolism through modulation of PPARα, FOXO3, and PGC1α [92–94]. In addition, SIRT6 controls circadian chromatin recruitment of SREBP-1 in the liver resulting in cyclic expression of genes involved in fatty acid and cholesterol metabolism, whilst SIRT3 regulates the rhythmic acetylation and activity of oxidative enzymes and respiration in mitochondria [95, 96].

Although most of the findings are in metabolic organs other than the heart, such as the liver and the white adipose tissue, NAD+ levels likely play similar roles in rhythmic metabolic changes in the heart, as NAD levels also oscillate in cardiomyocytes, and this oscillation is attenuated in CCM mice [97, 98]. Sirtuins also have roles in regulation of metabolism in the heart; loss of SIRT1 abolished the effects of PPARα on cardiomyocyte fatty acid oxidation, and SIRT3 interacts with acetyl-CoA synthetase 2, a cardiac-enriched mitochondrial enzyme promoting entry of acetyl-CoA into the TCA cycle [99, 100].

NAD+ as a cofactor serves as a potential link between the circadian clock and numerous metabolic enzymes in the heart. SDH and IDH3 are two intriguing examples. In addition to their cofactor levels, they also oscillate at the transcript level. SDH and IDH3 are critical enzymes in the TCA cycle and SDH is also complex II in the oxidative phosphorylation chain. Their rhythmicities highlight the importance of circadian regulation in oxidative metabolism in the heart; however, the exact evolutionary benefits of this additional layer of regulation are yet to be discovered. Furthermore, SIRT3 post-translationally modifies SDH, providing evidence of extensive cross-regulation among metabolic pathways; indeed, none of the above mechanisms ought to be considered in isolation [101].

Metabolism is a recurrent theme in circadian regulation. Elegant studies by Wang et al. have elucidated one logic of circadian regulation as a strategy to economize costly gene expression. The oscillatory genes tend to be highly expressed genes of the given tissue and through upregulating them at the required time and offsetting the peak with the trough, the overall energetic cost remains relatively low [102]. Thus, cyclic gene expression is intrinsically connected to energy utilization. As translation is costlier than transcription, it makes sense to exert control at the level of transcription. Direct comparison of the transcriptomic and proteomic dataset is required to further clarify this hypothesis.

Recent studies in mouse liver and drosophila head have demonstrated that only ~30% of the steady-state “oscillation” was a direct result of transcription [103, 104]. In the near future, it is imperative to use novel techniques such as nascent transcript RNAseq and RNA half-life measurements to further dissect the mechanisms by which precise circadian gene regulation is achieved.

### Proteomics

Proteomic studies are still limited by sensitivity; for example, in a transcriptomic study usually several thousand genes are identified as oscillatory, but only a few hundred may be identified in a proteomic study of the same tissue [105, 106]. However, proteomics offers information closer to functionality, as only about half of the oscillatory genes overlap when comparing transcriptomic and proteomic studies. Podobed et al. used two-dimensional gel electrophoresis and mass spectrometry to investigate the circadian proteomics of the heart and identified 8% (90/1147) of the soluble cardiac proteome as “oscillatory”. Importantly, they demonstrated that the CCM hearts had 4% (56/1471) of the soluble proteome which was differentially expressed from the wild type, including several key enzymes regulating multiple catabolic pathways such as mitochondrial pyruvate dehydrogenase E1a, aspartate aminotransferase, mitochondrial dihydrolipoyllysine succinyltransferase of 2-oxoglutarate dehydrogenase, aldehyde dehydrogenase, l-lactate dehydrogenase, enoyl-CoA hydratase, and d-β-hydroxybutyrate dehydrogenase [40]. This further strengthens previous observations from the transcriptomic studies suggesting the clock regulates catabolism to optimize active phase energy utilization.

Organelle level proteomics has greatly increased the sensitivity in quantifying low-abundance proteins. In the liver, almost 40% of the mitochondrial proteome and 10% of the nuclear proteome were found to oscillate in a circadian fashion [107, 108]. We look forward to similar studies in the heart.

### Post-translational modification

Protein O-linked β-*N*-acetylglucosamine (O-GlcNAc) is a reversible covalent post-translational modification catalyzed by O-GlcNAc transferase (OGT) using fructose 6-phosphate and glutamine as substrates and removed/hydrolyzed by O-GlcNAcase (OGA). It, therefore, may serve as a nutrient sensor for glucose and amino acid availability. Like phosphorylation, O-GlcNAcylation affects every aspect of its target protein, including subcellular localization, half-life, and activity [109–111]. Importantly, cardiac O-GlcNAc levels as well as O-GlcNac transferase OGT levels are circadian, observations which are not found in CCM hearts. Many important metabolic regulators are subjected to O-GlcNAc modification, such as AMPK, AKT, and GSK3β. Further, BMAL1 can be modified with O-GlcNAc and pharmacologically increasing cellular protein O-GlcNAcylation leads to induced BMAL1 mRNA and reduced PER2 protein, although the precise mechanism is yet to be elucidated [112].

Other nutrient-sensing transcriptional regulatory pathways and protein kinases are also potential candidates to bridge cellular metabolism to the core clock machinery. For example, 5′ AMP-activated protein kinase (AMPK) responds to AMP:ATP ratio and thus is a sensitive gauge for cellular energy status. AMPK activity in mouse livers is circadian and AMPK phosphorylates CRY1 to rhythmically alter CRY1 protein levels, providing a direct link between AMP/ATP ratio and the circadian clock [113]. In the heart, it has been observed that inhibition of hormone-sensitive lipase through AMPK-dependent phosphorylation has a diurnal variation, which was reduced in CCM mice, suggesting that AMP/ATP ratio remains intimately linked to the cardiomyocyte peripheral clock [98]. These molecular changes likely mediate the circadian variations in triglyceride turnover and lipolysis observed in WT mice and which are suppressed in CCM mice.

A recent report of phosphoproteomics in the liver demonstrated that at least a quarter of the phosphoproteome and more than 40% of the phosphoproteins are regulated in a circadian manner with large amplitude, which enables rapid and economic temporal regulation [114]. It would not be surprising if circadian regulation of the phosphoproteome also plays a significant role in the heart.

## Rhythmic cardiac metabolism

At rest, a healthy heart utilizes primarily fatty acids as substrates for energy, which is almost entirely through oxidative phosphorylation in the mitochondria [115]. In response to different physiologic and pathologic demands, the heart adapts its substrate use via a change in the relative contribution of substrates to energy metabolism. For example, under fasting conditions circulating levels of free fatty acids rise, leading to increased use by the heart as well as reciprocal reduced contribution by glucose, while during prolonged exercise, contribution by lactate, ketone bodies rise in addition to fatty acids [116, 117]. Therefore, although commonly termed a metabolic “omnivore”, in addition to tremendous energetic needs, the heart demonstrates significant plasticity in the fuels it devours.

The role of the peripheral cardiac clock in metabolism is supported through studies of its disruption in CCM mice, which show attenuated induction of myocardial fatty acid-responsive genes during fasting [11]. Moreover, in CCM hearts, myocardial oxygen consumption and fatty acid oxidation rates were increased and cardiac efficiency was decreased, without alterations in mitochondrial content or structure and only modest mitochondrial dysfunction [36].

**Fig. 4:**
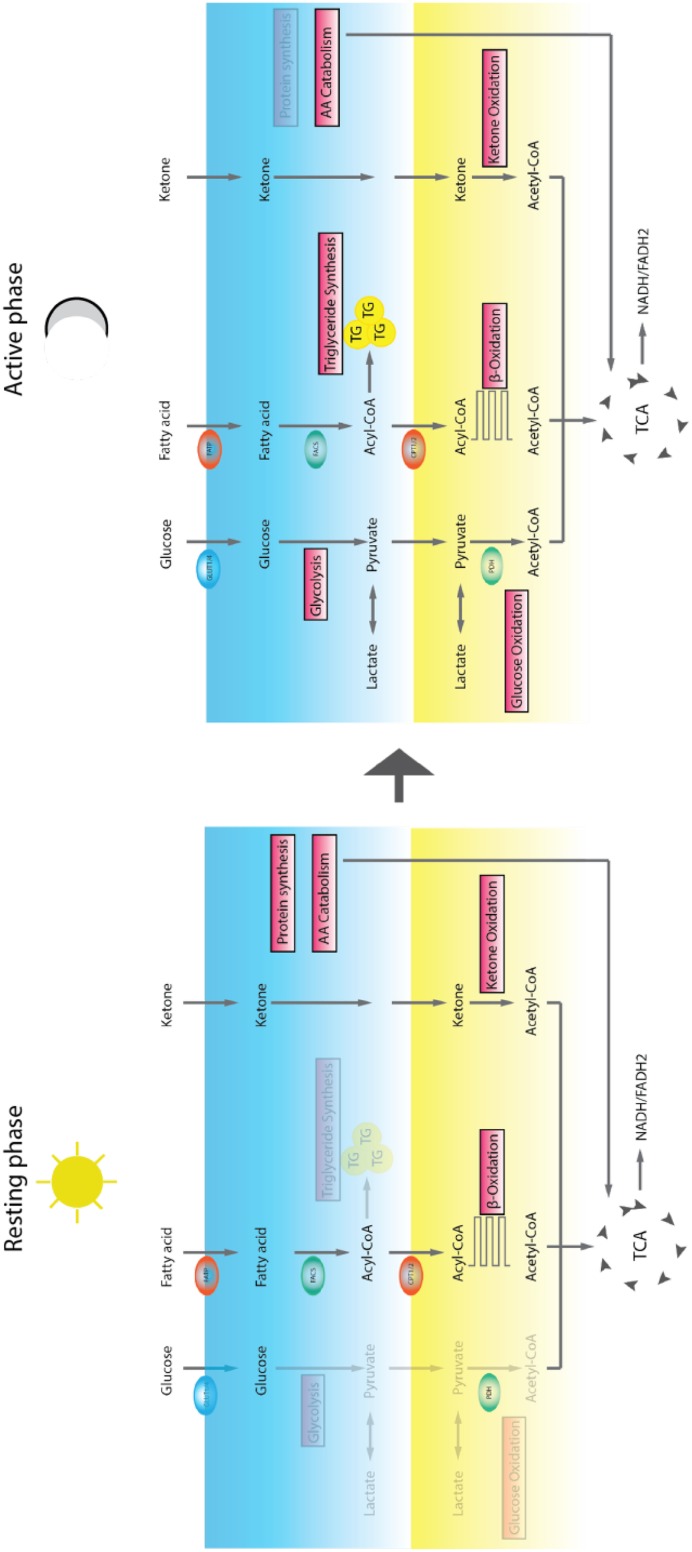
Metabolic flexibility and time-of-day alterations in substrate utilization in experimental mouse models. *TG* triglyceride

Fatty acid uptake in the heart is largely dependent on concentrations of circulating free fatty acids [118]. Thus, as fatty acid concentrations change, its rate of oxidation follows. During prolonged fasting and certain pathological states, such as metabolic heart disease and diabetes, free fatty acid concentrations rise as they are released from adipocytes, leading to increased fatty acid uptake and oxidation and depressed glucose oxidation [119]. The mechanisms by which this occurs have been well elucidated, whereby elevated fatty acid oxidation (and an elevation of free fatty acids in the circulation) inhibits the activity of phosphofructokinase-1 and -2 as well as pyruvate dehydrogenase [120, 121]. However, in perfused rat hearts harvested from rats at different time of the day, little variation in oleate oxidation is observed. This surprising results may represent differences between in vivo and ex vivo experiments, requiring further investigation (in vivo labeling studies) to clarify. Interestingly, triglyceride turnover exhibits ex vivo rhythmicity [122]. Triglyceride synthesis is increased during the awake period, when free fatty acids are low, while lipolysis is increased during the sleep phase, when free fatty acids are high [98]. These observations are postulated to cause recurrent time-of-day-dependent “decoupling” of triglyceride synthesis (antiphasic to availability) and fatty acid oxidation (phasic to availability). The mismatch in free fatty acid availability, storage, and use may lead to “lipotoxicity” (excess of free fatty acid) in the resting phase and decreased cardiac contractility due to the reduced efficiency of fatty acids as a substrate compared to glucose per molecule of oxygen required [123]. Indeed, increased oleate availability decreases myocardial contractile function most strongly during the sleep phase, presumably by exacerbating the mismatch (Fig. 4) [122].

In addition to the diurnal variation in fatty acid utilization, evidence suggests an increased utilization of glucose and lactate during the active phase, where exercise and food intake occur. In ex vivo experiments using perfused rat hearts Young et al. demonstrate a twofold increase in glucose oxidation during the active phase. In the same study, the investigators found that lactate oxidation in rat hearts was increased in the active phase, a finding they point out is consistent with a report that the lactate dehydrogenase A gene is induced during the onset of the dark phase [10, 124]. Additionally, under conditions of high workload, rates of lactate release measured in perfused mouse hearts were biphasic and highest in the active phase, while this biphasic behavior was abolished in the CCM mice [11]. This has been found to be true in human hearts as well, which preferentially utilize glucose and lactate during periods of moderately increased energy demand such as exercise or during certain pathologic states such as left ventricular hypertrophy, systemic hypertension, and myocardial ischemia [125, 126]. In a study of eleven athletes performing rowing exercises at different times of the day, blood lactate collected at the end of the endurance exercise oscillated over a 24-h period, although it remains unclear whether this is primarily due to contribution by the heart, or by the liver or the skeletal muscle metabolism; the respective contributions of these organs over the 24-h cycle to lactate levels in the serum require extensive investigation [127]. These findings together indicate that carbohydrate oxidation is likely utilized preferentially as workload increases during the awake phase due to its greater efficiency at a given rate of oxygen consumption (Fig. 4) [128].

Diurnal variations in amino acid metabolism in the heart remain an active area of investigation. Studies have shown that expression of amino acid catabolism enzymes is rhythmic in the heart and that protein synthesis is increased in the sleep phase [84, 85]. Additionally, as substrate availability is an important predictor for the uptake and use of that substrate by the heart, the fact that amino acid levels exhibit rhythmicity in the circulation would suggest a similar rhythmicity in their use in the heart (Fig. 4) [129, 130]. He et al. have recently demonstrated clock regulation of branched chain amino acid metabolism through a post-translational modification, biotinylation. In CCM and CBK mice, expression of the sodium-dependent multivitamin transporter (SMVT), which is responsible for biotin transport into cells, was reduced in a time-of-day-independent manner. Total protein biotinylation was also decreased, along with decreased fatty acid oxidation and leucine oxidation associated with decreased biotinylation of acetyl-CoA carboxylase and methylcrotonyl-CoA carboxylate, respectively. Importantly, these changes could be reversed by biotin supplementation [131].

Another major class of energetic substrates, ketones, is even less understood. There is little doubt that levels of circulating ketones can vary markedly given factors such as level of physical activity or prolonged fasting, and in both CCM and CBK mice ketone oxidation has been found to be decreased, likely due to decreased activity of β-hydroxybutyrate dehydrogenase 1 (Fig. 4) [86, 132].

## Conclusion

**Fig. 5:**
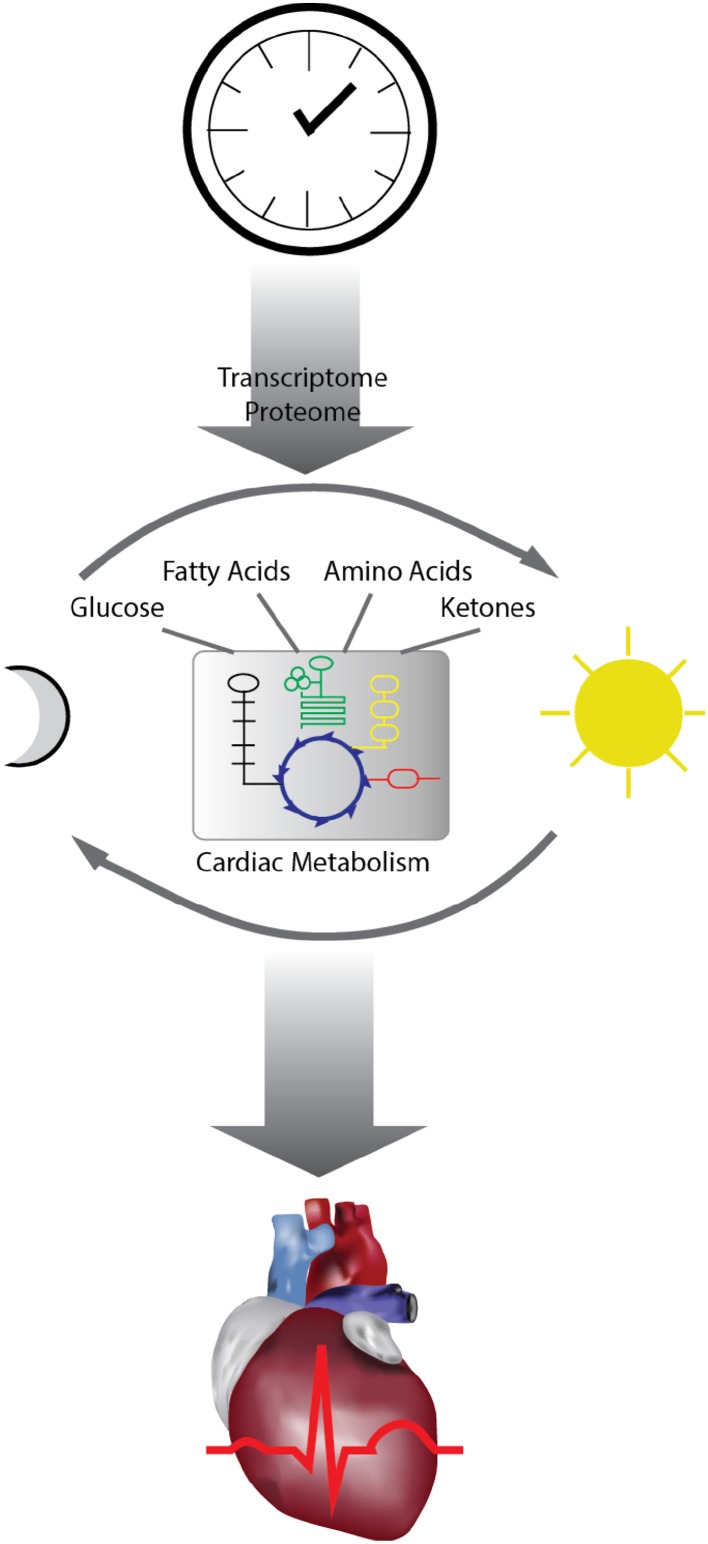
The peripheral cardiac clock coordinates rhythmic metabolism and diurnal cardiac physiology via key transcriptional outputs

Many of the observed diurnal variations in cardiac metabolism persist in ex vivo hearts, suggesting that they are mediated through intrinsic peripheral clock mechanisms. Transcriptomics and proteomics studies provide further evidence that diurnal variations occur at both RNA and protein levels of an array of metabolic genes which may mediate alterations in cardiac metabolism (Fig. 5) [40, 85]. Oscillatory NAD+ levels provide an additional link between the circadian clock and cellular metabolism through sirtuin activities and direct regulation of numerous metabolic enzymes. We are just beginning to understand the roles of other post-translational mechanisms in circadian regulation of metabolism. Our insights into the molecular link between the core clock and cardiac metabolism not only will improve our understanding of cardiac physiology and diseases, but also hold promise in providing novel therapeutic options in the future.

## References

[1] Stephan F, Zucker I (1972). Circadian rhythms in drinking behavior and locomotor activity of rats are eliminated by hypothalamic lesions. Proc Natl Acad Sci USA.

[2] Hastings M, Reddy A, Maywood E (2003). A clockwork web: circadian timing in brain and periphery, in health and disease. Nat Rev Neurosci.

[3] Damiola F (2000). Restricted feeding uncouples circadian oscillators in peripheral tissues from the central pacemaker in the suprachiasmatic nucleus. Genes Dev.

[4] Oishi K, Miyazaki K, Ishida N (2002). Functional CLOCK is not involved in the entrainment of peripheral clocks to the restricted feeding: entrainable expression of mPer2 and BMAL1 mRNAs in the heart of Clock mutant mice on Jcl:ICR background. Biochem Biophys Res Commun.

[5] Cailotto C (2009). Effects of nocturnal light on (clock) gene expression in peripheral organs: a role for the autonomic innervation of the liver. PLoS One.

[6] Stern N (1986). Circadian rhythm of plasma renin activity in older normal and essential hypertensive men: relation with inactive renin, aldosterone, cortisol and REM sleep. J Hypertens.

[7] Charloux A, Gronfier C, Lonsdorfer-Wolf E, Piquard F, Brandenberger G (1999). Aldosterone release during the sleep-wake cycle in humans. Am J Physiol.

[8] Watanabe T, Uchiyama Y (1988). Quantitative analyses of atrial myoendocrine cells and plasma atrial natriuretic peptides (ANP) of the rat with special reference to the twenty-four-hour variations in secretory granules and plasma ANP concentrations. Cell Tissue Res.

[9] Kretschmannova K, Svobodova I, Balik A, Mazna P, Zemkova H (2005). Circadian rhythmicity in AVP secretion and GABAergic synaptic transmission in the rat suprachiasmatic nucleus. Ann NY Acad Sci.

[10] Young M, Razeghi P, Cedars AM, Guthrie PH, Taegtmeyer H (2001). Intrinsic diurnal variations in cardiac metabolism and contractile function. Circ Res.

[11] Durgan DJ (2006). The circadian clock within the cardiomyocyte is essential for responsiveness of the heart to fatty acids. J Biol Chem.

[12] Millar-Craig MW, Bishop CN, Raftery EB (1978). Circadian variation of blood-pressure. Lancet.

[13] Huikuri HV (1992). Circadian rhythm of heart rate variability in survivors of cardiac arrest. Am J Cardiol.

[14] Willich SN (1987). Circadian variation in the incidence of sudden cardiac death in the Framingham Heart Study population. Am J Cardiol.

[15] Kaasik K, Lee CC (2004). Reciprocal regulation of haem biosynthesis and the circadian clock in mammals. Nature.

[16] Rutter J, Reick M, McKnight SL (2002). Metabolism and the control of circadian rhythms. Annu Rev Biochem.

[17] Tu BP, McKnight SL (2006). Metabolic cycles as an underlying basis of biological oscillations. Nat Rev Mol Cell Biol.

[18] Bass J, Takahashi JS (2010). Circadian integration of metabolism and energetics. Science.

[19] Takahashi JS, Hong HK, Ko CH, McDearmon EL (2008). The genetics of mammalian circadian order and disorder: implications for physiology and disease. Nat Rev Genet.

[20] Lowrey PL, Takahashi JS (2004). Mammalian circadian biology: elucidating genome-wide levels of temporal organization. Annu Rev Genom Hum Genet.

[21] Preitner N (2002). The orphan nuclear receptor REV-ERBalpha controls circadian transcription within the positive limb of the mammalian circadian oscillator. Cell.

[22] Guillaumond F, Dardente H, Giguere V, Cermakian N (2005). Differential control of Bmal1 circadian transcription by REV-ERB and ROR nuclear receptors. J Biol Rhythm.

[23] Papazyan R, Zhang Y, Lazar MA (2016). Genetic and epigenomic mechanisms of mammalian circadian transcription. Nat Struct Mol Biol.

[24] Etchegaray JP, Lee C, Wade PA, Reppert SM (2003). Rhythmic histone acetylation underlies transcription in the mammalian circadian clock. Nature.

[25] Ripperger JA, Schibler U (2006). Rhythmic CLOCK-BMAL1 binding to multiple E-box motifs drives circadian Dbp transcription and chromatin transitions. Nat Genet.

[26] Lee Y (2010). Coactivation of the CLOCK–BMAL1 complex by CBP mediates resetting of the circadian clock. J Cell Sci.

[27] Naruse Y (2004). Circadian and light-induced transcription of clock gene Per1 depends on histone acetylation and deacetylation. Mol Cell Biol.

[28] Curtis AM (2004). Histone acetyltransferase-dependent chromatin remodeling and the vascular clock. J Biol Chem.

[29] Feng D (2011). A circadian rhythm orchestrated by histone deacetylase 3 controls hepatic lipid metabolism. Science.

[30] Doi M, Hirayama J, Sassone-Corsi P (2006). Circadian regulator CLOCK is a histone acetyltransferase. Cell.

[31] Koike N (2012). Transcriptional architecture and chromatin landscape of the core circadian clock in mammals. Science.

[32] Meng QJ (2008). Setting clock speed in mammals: the CK1 epsilon tau mutation in mice accelerates circadian pacemakers by selectively destabilizing PERIOD proteins. Neuron.

[33] Siepka SM (2007). Circadian mutant overtime reveals F-box protein FBXL3 regulation of cryptochrome and period gene expression. Cell.

[34] Busino L (2007). SCFFbxl3 controls the oscillation of the circadian clock by directing the degradation of cryptochrome proteins. Science.

[35] Shea SA, Hilton MF, Hu K, Scheer FA (2011). Existence of an endogenous circadian blood pressure rhythm in humans that peaks in the evening. Circ Res.

[36] Durgan DJ (2011). Evidence suggesting that the cardiomyocyte circadian clock modulates responsiveness of the heart to hypertrophic stimuli in mice. Chronobiol Int.

[37] Collins HE, Rodrigo GC (2010). Inotropic response of cardiac ventricular myocytes to beta-adrenergic stimulation with isoproterenol exhibits diurnal variation: involvement of nitric oxide. Circ Res.

[38] Bray MS (2008). Disruption of the circadian clock within the cardiomyocyte influences myocardial contractile function, metabolism, and gene expression. Am J Physiol Heart Circ Physiol.

[39] Martino TA (2007). Disturbed diurnal rhythm alters gene expression and exacerbates cardiovascular disease with rescue by resynchronization. Hypertension.

[40] Podobed P (2014). The day/night proteome in the murine heart. Am J Physiol Regul Integr Comp Physiol.

[41] Clark LA (1987). A quantitative analysis of the effects of activity and time of day on the diurnal variations of blood pressure. J Chronic Dis.

[42] Fava C (2005). Dipping and variability of blood pressure and heart rate at night are heritable traits. Am J Hypertens.

[43] Hu K, Scheer FA, Buijs RM, Shea SA (2008). The circadian pacemaker generates similar circadian rhythms in the fractal structure of heart rate in humans and rats. Cardiovasc Res.

[44] Schroder EA (2013). The cardiomyocyte molecular clock, regulation of Scn5a, and arrhythmia susceptibility. Am J Physiol Cell Physiol.

[45] Li Y (2010). Prognostic value of the morning blood pressure surge in 5645 subjects from 8 populations. Hypertension.

[46] Zakopoulos NA (2005). Time rate of blood pressure variation is associated with increased common carotid artery intima-media thickness. Hypertension.

[47] Marfella R (2007). Morning blood pressure surge as a destabilizing factor of atherosclerotic plaque: role of ubiquitin–proteasome activity. Hypertension.

[48] Nguyen KD (2013). Circadian gene Bmal1 regulates diurnal oscillations of Ly6C(hi) inflammatory monocytes. Science.

[49] Scheiermann C (2012). Adrenergic nerves govern circadian leukocyte recruitment to tissues. Immunity.

[50] Keller M (2009). A circadian clock in macrophages controls inflammatory immune responses. Proc Natl Acad Sci USA.

[51] Schloss MJ (2016). The time-of-day of myocardial infarction onset affects healing through oscillations in cardiac neutrophil recruitment. EMBO Mol Med.

[52] Jovicic A, Ivanisevic V, Nikolajevic R (1991). Circadian variations of platelet aggregability and fibrinolytic activity in patients with ischemic stroke. Thromb Res.

[53] Undar L, Turkay C, Korkmaz L (1989). Circadian variation in circulating platelet aggregates. Ann Med.

[54] Pinotti M (2005). Daily and circadian rhythms of tissue factor pathway inhibitor and factor VII activity. Arterioscler Thromb Vasc Biol.

[55] Kapiotis S (1997). Morning hypercoagulability and hypofibrinolysis. Diurnal variations in circulating activated factor VII, prothrombin fragment F1 + 2, and plasmin-plasmin inhibitor complex. Circulation.

[56] Tofler GH (1987). Concurrent morning increase in platelet aggregability and the risk of myocardial infarction and sudden cardiac death. N Engl J Med.

[57] Rosing DR (1970). Blood fibrinolytic activity in man. Diurnal variation and the response to varying intensities of exercise. Circ Res.

[58] Angleton P, Chandler WL, Schmer G (1989). Diurnal variation of tissue-type plasminogen activator and its rapid inhibitor (PAI-1). Circulation.

[59] Thogersen AM (1998). High plasminogen activator inhibitor and tissue plasminogen activator levels in plasma precede a first acute myocardial infarction in both men and women: evidence for the fibrinolytic system as an independent primary risk factor. Circulation.

[60] Muller JE (1985). Circadian variation in the frequency of onset of acute myocardial infarction. N Engl J Med.

[61] Mogabgab O (2013). Relation between time of symptom onset of ST-segment elevation myocardial infarction and patient baseline characteristics: from the National Cardiovascular Data Registry. Clin Cardiol.

[62] Cannon CP (1997). Circadian variation in the onset of unstable angina and non-Q-wave acute myocardial infarction (the TIMI III Registry and TIMI IIIB). Am J Cardiol.

[63] Ridker PM, Manson JE, Buring JE, Muller JE, Hennekens CH (1990). Circadian variation of acute myocardial infarction and the effect of low-dose aspirin in a randomized trial of physicians. Circulation.

[64] Durgan DJ (2010). Short communication: ischemia/reperfusion tolerance is time-of-day-dependent: mediation by the cardiomyocyte circadian clock. Circ Res.

[65] Eckle T (2012). Adora2b-elicited Per2 stabilization promotes a HIF-dependent metabolic switch crucial for myocardial adaptation to ischemia. Nat Med.

[66] Virag JA (2010). Attenuation of myocardial injury in mice with functional deletion of the circadian rhythm gene mPer2. Am J Physiol Heart Circ Physiol.

[67] Virag JA (2013). Cardioprotection via preserved mitochondrial structure and function in the mPer2-mutant mouse myocardium. Am J Physiol Heart Circ Physiol.

[68] Rotter D (2014). Calcineurin and its regulator, RCAN1, confer time-of-day changes in susceptibility of the heart to ischemia/reperfusion. J Mol Cell Cardiol.

[69] Reiter R, Swingen C, Moore L, Henry TD, Traverse JH (2012). Circadian dependence of infarct size and left-ventricular function following ST-elevation myocardial infarction. Circ Res.

[70] Suarez-Barrientos A (2011). Circadian variations of infarct size in acute myocardial infarction. Heart (British Cardiac Society).

[71] Arroyo Ucar E, Dominguez-Rodriguez A, Abreu-Gonzalez P (2012). Influence of diurnal variation in the size of acute myocardial infarction. Med Intensiva.

[72] Fournier S (2012). Circadian variations of ischemic burden among patients with myocardial infarction undergoing primary percutaneous coronary intervention. Am Heart J.

[73] Fournier S (2015). Myocardial infarct size and mortality depend on the time of day—a large multicenter study. PLoS One.

[74] Bulluck H (2017). Circadian variation in acute myocardial infarct size assessed by cardiovascular magnetic resonance in reperfused STEMI patients. Int J Cardiol.

[75] Seneviratna A (2015). Circadian dependence of infarct size and acute heart failure in ST elevation myocardial infarction. PLoS One.

[76] Zhang R, Lahens NF, Ballance HI, Hughes ME, Hogenesch JB (2014). A circadian gene expression atlas in mammals: Implications for biology and medicine. Proc Natl Acad Sci USA.

[77] Hermida RC, Ayala DE, Calvo C, Lopez JE (2005). Aspirin administered at bedtime, but not on awakening, has an effect on ambulatory blood pressure in hypertensive patients. J Am Coll Cardiol.

[78] Hermida RC (2005). Differing administration time-dependent effects of aspirin on blood pressure in dipper and non-dipper hypertensives. Hypertension.

[79] Hermida RC, Ayala DE (2009). Chronotherapy with the angiotensin-converting enzyme inhibitor ramipril in essential hypertension: improved blood pressure control with bedtime dosing. Hypertension.

[80] Sleight P (2001). Blood-pressure reduction and cardiovascular risk in HOPE study. Lancet.

[81] Flack JM, Nasser SA (2011). Benefits of once-daily therapies in the treatment of hypertension. Vasc Health Risk Manag.

[82] Hermida RC, Ayala DE, Mojon A, Fernandez JR (2010). Influence of circadian time of hypertension treatment on cardiovascular risk: results of the MAPEC study. Chronobiol Int.

[83] Panda S (2002). Coordinated transcription of key pathways in the mouse by the circadian clock. Cell.

[84] Storch KF (2002). Extensive and divergent circadian gene expression in liver and heart. Nature.

[85] Zhang L (2015). KLF15 establishes the landscape of diurnal expression in the heart. Cell Rep.

[86] Young ME (2014). Cardiomyocyte-specific BMAL1 plays critical roles in metabolism, signaling, and maintenance of contractile function of the heart. J Biol Rhythm.

[87] Jeyaraj D (2012). Circadian rhythms govern cardiac repolarization and arrhythmogenesis. Nature.

[88] Nakahata Y, Sahar S, Astarita G, Kaluzova M, Sassone-Corsi P (2009). Circadian control of the NAD+ salvage pathway by CLOCK-SIRT1. Science.

[89] Ramsey KM (2009). Circadian clock feedback cycle through NAMPT-mediated NAD+ biosynthesis. Science.

[90] Nakahata Y (2008). The NAD+-dependent deacetylase SIRT1 modulates CLOCK-mediated chromatin remodeling and circadian control. Cell.

[91] Asher G (2008). SIRT1 regulates circadian clock gene expression through PER2 deacetylation. Cell.

[92] Rodgers JT (2005). Nutrient control of glucose homeostasis through a complex of PGC-1alpha and SIRT1. Nature.

[93] Brunet A (2004). Stress-dependent regulation of FOXO transcription factors by the SIRT1 deacetylase. Science.

[94] Purushotham A (2009). Hepatocyte-specific deletion of SIRT1 alters fatty acid metabolism and results in hepatic steatosis and inflammation. Cell Metab.

[95] Masri S (2014). Partitioning circadian transcription by SIRT6 leads to segregated control of cellular metabolism. Cell.

[96] Peek CB (2013). Circadian clock NAD+ cycle drives mitochondrial oxidative metabolism in mice. Science.

[97] Powanda MC, Wannemacher RW (1970). Evidence for a linear correlation between the level of dietary tryptophan and hepatic NAD concentration and for a systematic variation in tissue NAD concentration in the mouse and the rat. J Nutr.

[98] Tsai JY (2010). Direct regulation of myocardial triglyceride metabolism by the cardiomyocyte circadian clock. J Biol Chem.

[99] Planavila A, Iglesias R, Giralt M, Villarroya F (2011). Sirt1 acts in association with PPARα to protect the heart from hypertrophy, metabolic dysregulation, and inflammation. Cardiovasc Res.

[100] Hallows WC, Lee S, Denu JM (2006). Sirtuins deacetylate and activate mammalian acetyl-CoA synthetases. Proc Natl Acad Sci USA.

[101] Cimen H (2010). Regulation of succinate dehydrogenase activity by SIRT3 in mammalian mitochondria. Biochemistry.

[102] Wang GZ (2015). Cycling transcriptional networks optimize energy utilization on a genome scale. Cell Rep.

[103] Rodriguez J (2013). Nascent-Seq analysis of Drosophila cycling gene expression. Proc Natl Acad Sci USA.

[104] Menet JS, Rodriguez J, Abruzzi KC, Rosbash M (2012). Nascent-Seq reveals novel features of mouse circadian transcriptional regulation. Elife.

[105] Mauvoisin D (2014). Circadian clock-dependent and -independent rhythmic proteomes implement distinct diurnal functions in mouse liver. Proc Natl Acad Sci USA.

[106] Robles MS, Cox J, Mann M (2014). In-vivo quantitative proteomics reveals a key contribution of post-transcriptional mechanisms to the circadian regulation of liver metabolism. PLoS Genet.

[107] Wang J (2017). Nuclear proteomics uncovers diurnal regulatory landscapes in mouse liver. Cell Metab.

[108] Neufeld-Cohen A (2016). Circadian control of oscillations in mitochondrial rate-limiting enzymes and nutrient utilization by PERIOD proteins. Proc Natl Acad Sci USA.

[109] Hanover JA, Krause MW, Love DC (2010). The hexosamine signaling pathway: O-GlcNAc cycling in feast or famine. Biochim Biophys Acta.

[110] Hart GW, Slawson C, Ramirez-Correa G, Lagerlof O (2011). Cross talk between O-GlcNAcylation and phosphorylation: roles in signaling, transcription, and chronic disease. Annu Rev Biochem.

[111] Kamemura K, Hart GW (2003). Dynamic interplay between O-glycosylation and O-phosphorylation of nucleocytoplasmic proteins: a new paradigm for metabolic control of signal transduction and transcription. Prog Nucleic Acid Res Mol Biol.

[112] Durgan DJ (2011). O-GlcNAcylation, novel post-translational modification linking myocardial metabolism and cardiomyocyte circadian clock. J Biol Chem.

[113] Lamia KA (2009). AMPK regulates the circadian clock by cryptochrome phosphorylation and degradation. Science.

[114] Robles MS, Humphrey SJ, Mann M (2017). Phosphorylation is a central mechanism for circadian control of metabolism and physiology. Cell Metab.

[115] Stanley WC, Recchia FA, Lopaschuk GD (2005). Myocardial substrate metabolism in the normal and failing heart. Physiol Rev.

[116] Taegtmeyer H (1994). Energy metabolism of the heart: from basic concepts to clinical applications. Curr Probl Cardiol.

[117] Opie LH, Evans JR, Shipp JC (1963). Effect of fasting on glucose and palmitate metabolism of perfused rat heart. Am J Physiol.

[118] Bing RJ, Siegel A, Ungar I, Gilbert M (1954). Metabolism of the human heart. II. Studies on fat, ketone and amino acid metabolism. Am J Med.

[119] Carvajal K, Moreno-Sanchez R (2003). Heart metabolic disturbances in cardiovascular diseases. Arch Med Res.

[120] Garland PB, Randle PJ, Newsholme EA (1963). Citrate as an intermediary in the inhibition of phosphofructokinase in rat heart muscle by fatty acids, ketone bodies, pyruvate, diabetes, and starvation. Nature.

[121] Randle PJ, Garland PB, Hales CN, Newsholme EA (1963). The glucose fatty-acid cycle. Its role in insulin sensitivity and the metabolic disturbances of diabetes mellitus. Lancet.

[122] Durgan DJ (2007). Circadian rhythms in myocardial metabolism and contractile function: influence of workload and oleate. Am J Physiol Heart Circ Physiol.

[123] Pascual F, Coleman RA (2016). Fuel availability and fate in cardiac metabolism: a tale of two substrates. Biochim Biophys Acta.

[124] Rutter J, Reick M, Wu LC, McKnight SL (2001). Regulation of clock and NPAS2 DNA binding by the redox state of NAD cofactors. Science.

[125] Gertz EW, Wisneski JA, Stanley WC, Neese RA (1988). Myocardial substrate utilization during exercise in humans. Dual carbon-labeled carbohydrate isotope experiments. J Clin Investig.

[126] Kemppainen J (2002). Myocardial and skeletal muscle glucose uptake during exercise in humans. J Physiol.

[127] Forsyth JJ, Reilly T (2004). Circadian rhythms in blood lactate concentration during incremental ergometer rowing. Eur J Appl Physiol.

[128] Burkhoff D (1991). Influence of metabolic substrate on rat heart function and metabolism at different coronary flows. Am J Physiol.

[129] Jeyaraj D (2012). Klf15 orchestrates circadian nitrogen homeostasis. Cell Metab.

[130] Eckel-Mahan KL (2012). Coordination of the transcriptome and metabolome by the circadian clock. Proc Natl Acad Sci USA.

[131] He L (2016). Biotinylation: a novel posttranslational modification linking cell autonomous circadian clocks with metabolism. Am J Physiol Heart Circ Physiol.

[132] Askew EW, Dohm GL, Huston RL (1975). Fatty acid and ketone body metabolism in the rat: response to diet and exercise. J Nutr.

